# Case report: Management of liver cancer complicated by gastric varices rupture and bleeding: transjugular intrahepatic portosystemic shunt utilizing the mesenteric venous pathway

**DOI:** 10.3389/fmed.2024.1388584

**Published:** 2024-06-19

**Authors:** Guoliang Sun, Jiangye Wang, Beining Zhang, Ninggang Zheng

**Affiliations:** The Department of Interventional Oncology, Gansu Provincial Hospital, Lanzhou, China

**Keywords:** TIPS, interventional, portal hypertension, portal vein thrombosis, hepatocellular carcinoma

## Abstract

To avoid recurrent variceal bleeding, transjugular intrahepatic portosystemic shunt (TIPS) in conjunction with variceal embolization is considered to be an effective strategy. However, due to changes in conditions and variations in the patient's state, individuals undergoing TIPS may face challenges and limitations during procedures. The transjugular technique and combined transsplenic portal venous recanalization (PVR) with TIPS were not effective in this case due to a blocked portal vein and a previous splenectomy. With an abdominal incision, we successfully punctured the mesenteric venous system and navigated the occluded segment of the portal vein through the mesenteric approach. TIPS was then performed under balloon guidance. This study aims to explore the management of risks and complications during surgical operations and propose multiple preoperative surgical techniques to improve the success rate of the procedure.

## 1 Introduction

Decompensated liver cirrhosis can result in severe vertical hemorrhage, with high mortality rates despite recent improvements in survival. A multidisciplinary approach is necessary to treat gastrointestinal bleeding in patients with advanced liver cirrhosis (1). Various therapies are used to prevent and manage shock, with additional goals of resuscitation and supportive care (2). Advanced liver cirrhosis requires a multidisciplinary approach to improve survival rates (3).

Persistent portal vein thrombosis (PVT) poses significant challenges, especially in patients with hepatocellular carcinoma (HCC) and a history of splenectomy. Transjugular intrahepatic portosystemic shunt (TIPS) is often utilized to reduce portal hypertension and manage variceal bleeding effectively. Despite technical difficulties and potential complications, TIPS remains a viable option for PVT treatment (4–6). However, the presence of PVT complicates conventional TIPS procedures as the occlusion of the portal vein limits the pathways for puncture and stent placement. Qi et al. (7) highlighted several approaches for TIPS placement in PVT patients, including transjugular, transhepatic, transsplenic, and transmesenteric methods. Although the mesenteric approach is less frequently used due to its specialized nature and higher risk, it becomes crucial when other routes are not feasible. Rossle et al., Blum et al., and Edelson et al. (8–10) emphasized the importance of TIPS in managing variceal bleeding and its complications. Despite certain complications such as hepatic encephalopathy, these are usually manageable. Literature reviews indicate that the long-term outcomes of TIPS are favorable, even with higher rates of stenosis and occlusion, as early detection and intervention can effectively prevent severe consequences. Bilbao et al. (11) explored the limitations of percutaneous methods in treating PVT, including fibrinolytic infusion, balloon angioplasty, and stent placement. Their studies showed that while these methods achieved clinical improvement in some acute PVT cases, the morphological results were not always ideal, with some patients experiencing re-thrombosis. This underscores the importance of selecting appropriate treatment methods in complex PVT cases. Rozenblit et al. (12) and Rozenblit et al. (13) proposed a combined transmesenteric and transfemoral approach for TIPS placement, integrating radiological and surgical techniques. They demonstrated successful cases of accessing the portal vein system through a small incision in the mesenteric vein in complex cases. This method improves instrument control and surgical efficiency, thereby reducing operation time and radiation exposure while providing better portal system evaluation. Entezari et al. (14), Dewald et al. (15), and Chamsuddin et al. (16) further expanded this technique by introducing a combined transmesenteric and transjugular approach (Meso-TIPS) for treating complex PVT cases, proving its efficacy and safety.

In our case report, a patient with decompensated liver cirrhosis, HCC, and a history of splenectomy presented with persistent PVT. Given the extensive thrombosis and exclusion of splenic access, the mesenteric route was the only viable option for TIPS placement. This method, underscores the feasibility and safety of the mesenteric pathway in complex PVT cases, aligning with the findings of previous studies (7, 12–16). The successful implementation of TIPS through the mesenteric route in this patient demonstrates a crucial therapeutic strategy for managing severe portal hypertension and its associated complications. This study emphasizes the importance of selecting appropriate access routes and using advanced imaging and thrombectomy techniques in managing complex PVT cases. By applying these advanced techniques to our case report, we highlight the reliability and efficacy of the mesenteric approach for TIPS. This approach not only addresses major clinical challenges but also improves the patient's long-term prognosis, thereby rendering our treatment strategy more credible and successful.

## 2 Case report

Patient History: The patient has a history of liver cirrhosis, hepatitis B, and a splenectomy performed in 2012. In April 2023, HCC was detected, accompanied by PVT, hypertension, and alterations in the portal vein. The initial Barcelona Clinic Liver Cancer (BCLC) staging was B-C due to thrombus uncertainty and carcinoma concerns. Before admission, the patient experienced gastrointestinal bleeding. On 13 November, 2023, varices were detected during an esophagogastroduodenoscopy. On 16 November, 2023, an enhanced CT scan showed no significant changes in the tumor compared to the initial CT scan. However, abnormal perfusion patterns were seen around it, mostly in liver segments I, VI, and VII (see Figure 2A). The liver is affected by cirrhosis, and the spleen has been removed. The portal vein is occluded and demonstrates fibrosis, with collateral circulation present. Additionally, there is mild intrahepatic bile duct dilation and fluid accumulation in the abdomen (see Figure 2B).

A multidisciplinary discussion ruled out band ligation for variceal bleeding due to its risk and ineffectiveness. Consequently, a TIPS procedure was selected as an alternative intervention. Initially, attempts through the jugular vein pathway failed, leading to the involvement of the surgery and anesthesia departments to execute the modified TIPS strategy. An 8-mm Viatorr-coated stent (W. L. Gore & Associates, Flagstaff, Arizona, USA) was used to create a shunt channel extending from the proximal hepatic vein to the main portal vein. Considering the patient's long-standing PVT and local narrowing of the mesenteric veins, the stent placement area was extended during the procedure using an 8-mm smart bare metal stent (Cordis Corporation, Milpitas, California, USA). Throughout the procedure, the partial pressure gradient (PPG) remained below 10 mm Hg. Prior to the TIPS development, the PPG was 10 mm Hg. The Viatorr TIPS-covered stent (7 cm polytetrafluoroethylene cover and 2 cm bare metal segment) was inserted into the portal vein, dilated to 8 mm, reducing the PPG to 4 mmHg. The stent's upper end (6-cm bare metal stent) extended to the border between the liver and the heart. The intestinal mesenteric thrombus was cleared during the procedure, with no persistent thrombosis detected. Subsequent angiography showed significant local mesenteric vein stenosis. To prevent postoperative thrombosis and associated complications, the narrowed segment was dilated with a balloon, and an 8-cm bare metal smart stent was placed to restore potency in the narrowed area of the mesenteric vein, as shown in the picture. After surgery, the patient developed pneumonia. A chest X-ray on 29 November 2023, 2023 showed bilateral inflammatory infiltrates with slight left lower lung field progression since 22 November, 2023. The patient's condition improved with treatment. Subsequent CT scans showed favorable outcomes without signs of gastrointestinal bleeding or hepatic encephalopathy.

## 3 Discussion

Patients with decompensated liver cirrhosis and malignant liver tumors, particularly those experiencing gastric variceal bleeding, face a poor prognosis. As discussed in the introduction, gastric variceal bleeding is a leading cause of mortality, primarily due to recurrent bleeding and liver failure (17). The coexistence of HCC, portal hypertension, and bleeding gastric varices complicates TIPS procedures. As highlighted in the introduction, TIPS is effective in managing clinically significant portal hypertension (CSPH) and HCC, with high success rates and minimal complications (18). Studies have demonstrated that combining TIPS with local treatments can prolong survival in HCC patients with portal hypertension (19). This aligns with the introduction's discussion on the multidisciplinary approach required for effective management of advanced liver cirrhosis. TIPS is a safe and effective method for HCC patients with symptomatic portal hypertension. TIPS should be considered for cases of HCC, especially for managing variceal bleeding or as a temporary measure in patients who are unable to undergo HCC treatment due to ascites or portal hypertension. TIPS can still be used for individuals with advanced liver cirrhosis and other health problems, even if they are also diagnosed with HCC.

**Figure 1:**
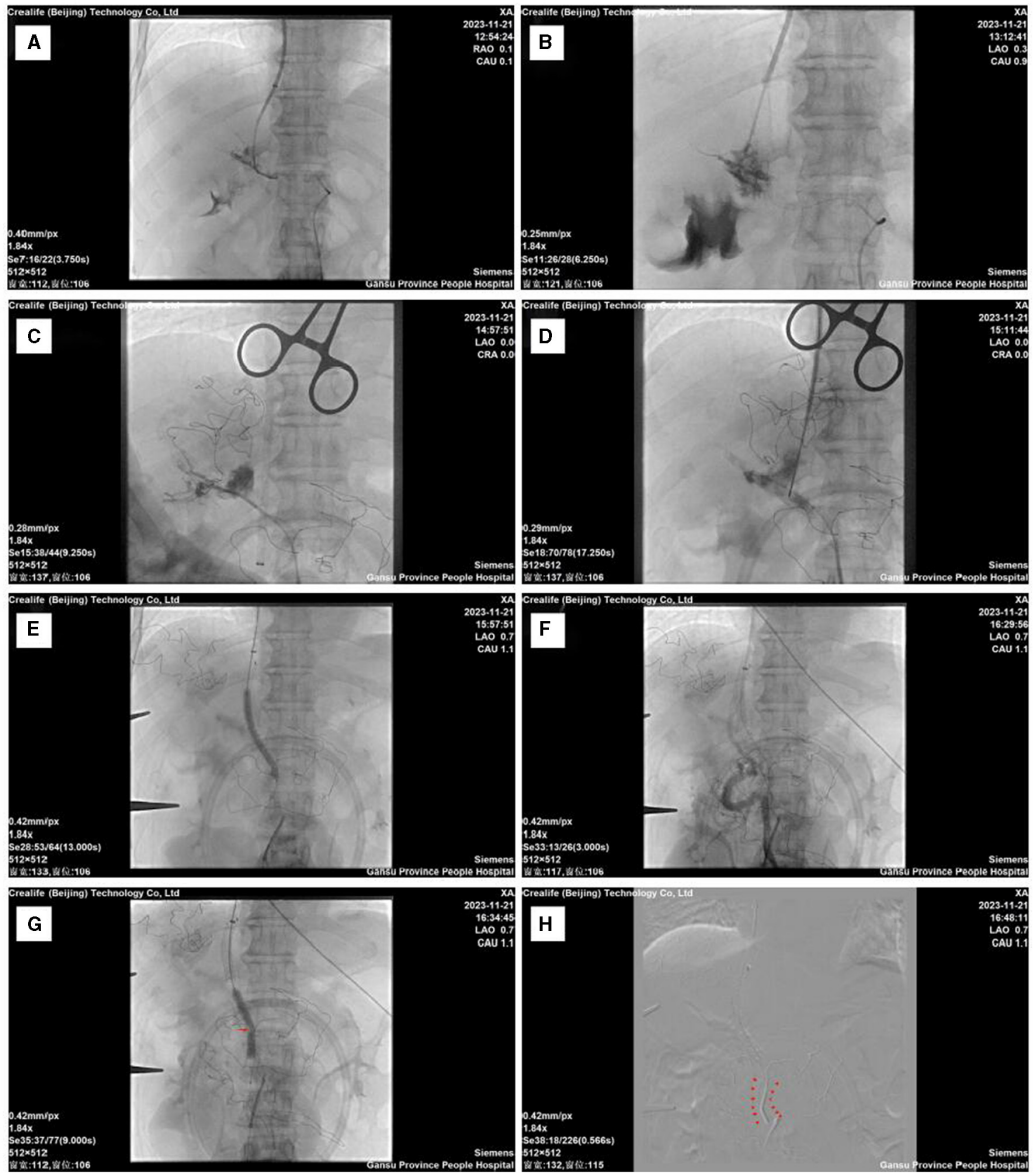
Intraoperative imaging data. **(A)** Successful puncture through the conventional approach. **(B)** Local rupture of the portal vein after multiple punctures. **(C)** Guidewire-assisted catheterization through the mesenteric route into the right branch of the portal vein. **(D)** Balloon-guided puncture technique. **(E)** Balloon dilation of the puncture tract and placement of the stent, followed by local balloon dilation within the stent. **(F)** Post-stent placement angiography reveals mesenteric thrombus. **(G)** Mesenteric venous stenosis balloon angioplasty. **(H)** Extended placement area of the mesenteric stent.

Initially, the conventional TIPS approach was considered but was not successful. Fluoroscopic imaging was used to confirm entry into the portal vein (Figure 1A), but the shunt channel could not be created. There was a local rupture and bleeding of the portal vein during the surgery (Figure 1B). This difficulty highlights the challenges mentioned in the introduction about the complexity of PVT in TIPS procedures. Multiple attempts to create a shunt channel through the thrombotic segment were unsuccessful, likely due to the chronic nature of the disease. Consequently, conventional interventional techniques were discontinued to minimize additional risks.

Placing a TIPS is particularly challenging in cases of splenic and portal vein obstructions, increasing the risk of complications and procedural complexity. This difficulty was anticipated in the introduction, which discusses the limitations of conventional access routes in the presence of extensive PVT. However, literature reviews, such as the one by Yan et al., unanimously conclude that TIPS for PVT is feasible and safe (7, 20). Portal vein obstruction should not be viewed as an absolute contraindication for TIPS (21). This is in line with the assertion made in the introduction that despite complications, TIPS remains a viable option for managing variceal bleeding and portal hypertension. TIPS procedures have been successfully performed in patients with portal vein occlusion due to thrombosis. TIPS has shown good efficacy in cases involving PVT (4, 22). The patient, experiencing severe portal vein blockage, underwent a splenectomy before the TIPS procedure. This necessitated ruling out traditional routes such as transjugular or splenic veins. The theoretical advantages of TIPS for PVT are deemed substantial, given its capacity for vascular restoration and prevention of thrombotic events and its associated complications through enhanced portal vein hemodynamics (23, 24). Different strategies and techniques have been developed to tackle challenges posed by chronic portal vein and splenic vein blockages. For PVR-TIPS, where a balloon-assisted shunt is created in the portal vein, ultrasound can guide a needle through the superior mesenteric vein (SMV) when direct liver or spleen access is not possible (15). The transjugular approach can cause problems during TIPS procedures. To avoid these problems, both the mesenteric and transjugular venous pathways can be used at the same time (14). This method has been shown to be feasible and effective for individuals with difficult splenic vein access (12, 13). It did not cause any acute issues, and follow-up imaging demonstrated positive outcomes with the placement of an intrahepatic portosystemic shunt (IPS) (25).

After thorough planning, a surgical strategy was devised. Transitioning swiftly to our backup plan when traditional methods failed, we encountered challenges with ultrasound-guided punctures on the mesenteric vein, specialized equipment needs, and limited expertise, prompting us to halt this method. Instead, we opted for the MAT (Mesenteric Approach Technique) method for performing TIPS with assistance from a small incision under general anesthesia. MAT-TIPS has also been successfully used in cirrhotic patients whose portal vein is completely blocked due to chronic thrombosis. This method presents a novel approach to conduct TIPS in these situations (26). It has been shown to have similar effects on intrahepatic shunt function and to cause similar complications during surgery as the standard transjugular method (27). The second surgical approach entailed higher risks and increased complexity. Despite encountering challenges, the procedure was successful. A small incision was made in the abdomen to access the abdominal cavity. Contrary to the intended target of the SMV, the inferior mesenteric vein was inadvertently punctured. Nevertheless, the surgery proceeded smoothly, and the catheter successfully reached the portal vein (Figure 1C). A balloon-assisted puncture technique was employed to facilitate the advancement of the balloon into the portal vein. The puncture needle was navigated through the jugular vein and directed toward the balloon for insertion. Subsequently, a guidewire was threaded through the balloon catheter and carefully maneuvered across the thrombotic segment (Figure 1D). The surgical plan, including stent placement and balloon dilation, was followed step by step (Figure 1E). Everything proceeded as planned.

First, we placed a Viatorr-coated stent within the established TIPS channel, navigating through the occluded segment of the portal vein. Immediate local contrast imaging revealed some thrombus formation in the mesenteric veins (Figure 1F). In this study, several factors were identified to be increasing the likelihood of acute mesenteric venous thrombosis (MVT), including smoking, high blood pressure, peritonitis, hemoglobin levels, albumin levels, intraperitoneal free fluid, decreased intestinal wall enhancement, and bowel distension (28). Additionally, factors such as a history of blood clotting problems, splenectomy, and symptoms like nausea, vomiting, abdominal pain, tenderness, and distension, along with lab results showing high plasma lactate levels and white blood cell counts, must be considered (29). The patient's history and examination results indicated several of these risk factors, including intra-abdominal fluid accumulation, hypoalbuminemia, and anemia. The patient's prior splenectomy further contributed to the development of MVT. Several factors influence the prognosis of acute MVT. However, early diagnosis and anticoagulant therapy can lead to rapid improvement in clinical symptoms (30). In cases of trauma-related MVT, standalone anticoagulation therapy may suffice if early recognition and treatment are feasible (31). Successful surgical intervention can result in favorable outcomes and recovery (32). In our case, we detected and confirmed the thrombosis during surgery using angiography. After three catheter-based aspirations, the majority of the thrombus was effectively cleared. Anticoagulant therapy was then initiated, and follow-up assessments indicated no thrombus-related risks or complications. Intraoperative vascular imaging showed severe narrowing in one area of the mesenteric veins after the thrombus was removed (Figure 1G). To prevent postoperative thrombus formation and secondary issues within the stent, we performed balloon dilation at the site of mesenteric venous stenosis. However, post-dilation imaging showed incomplete resolution of the stenosis problem. When the main portal vein or the upper mesenteric veins cannot be accessed during TIPS procedures, an extended stent should be used due to frequent blockages in the portal and splenic veins (15). It has been documented that extending stents to the distal SMV in TIPS procedures may jeopardize transplant surgeries(33). Therefore, the extension of TIPS stents to the SMV remains a topic of debate (34). In this case, we placed an uncovered stent at the distal end to ensure smooth blood flow through the narrowed segment of the mesenteric vein (Figure 1H). Contrast imaging showed effective blood flow through the TIPS channel into the heart, normalizing the portal pressure gradient. These results reinforce the introduction's insights on the efficacy of TIPS in managing portal hypertension despite complex challenges.

Postoperatively, the patient experienced occasional black stools, which were managed through the administration of acid-suppressing medications, fluid replacement, and the implementation of strict fasting policies. The black stools resolved, subsequent tests were negative for occult blood, and there was no notable drop in hemoglobin levels. Bleeding and potential mesenteric vein damage are risks following TIPS procedures. Patients with portal hypertension are more likely to experience these consequences due to their stronger vascular walls and higher portal vein pressure. Based on the patient's condition and the data we have collected, chronic liver disease is the primary cause of postoperative bleeding, while long-term portal hypertension and compromised vascular quality may contribute to the occurrence of black stools after surgery. In conclusion, our successful management of a complex case involving liver cancer, gastric varices, and portal venous cavernous transformation using a mesenteric approach for TIPS underscores the importance of selecting appropriate access routes and employing advanced imaging and thrombectomy techniques. This approach addresses major clinical challenges, improves patient prognosis, and highlights the reliability and efficacy of the mesenteric pathway for TIPS.

**Figure 2:**
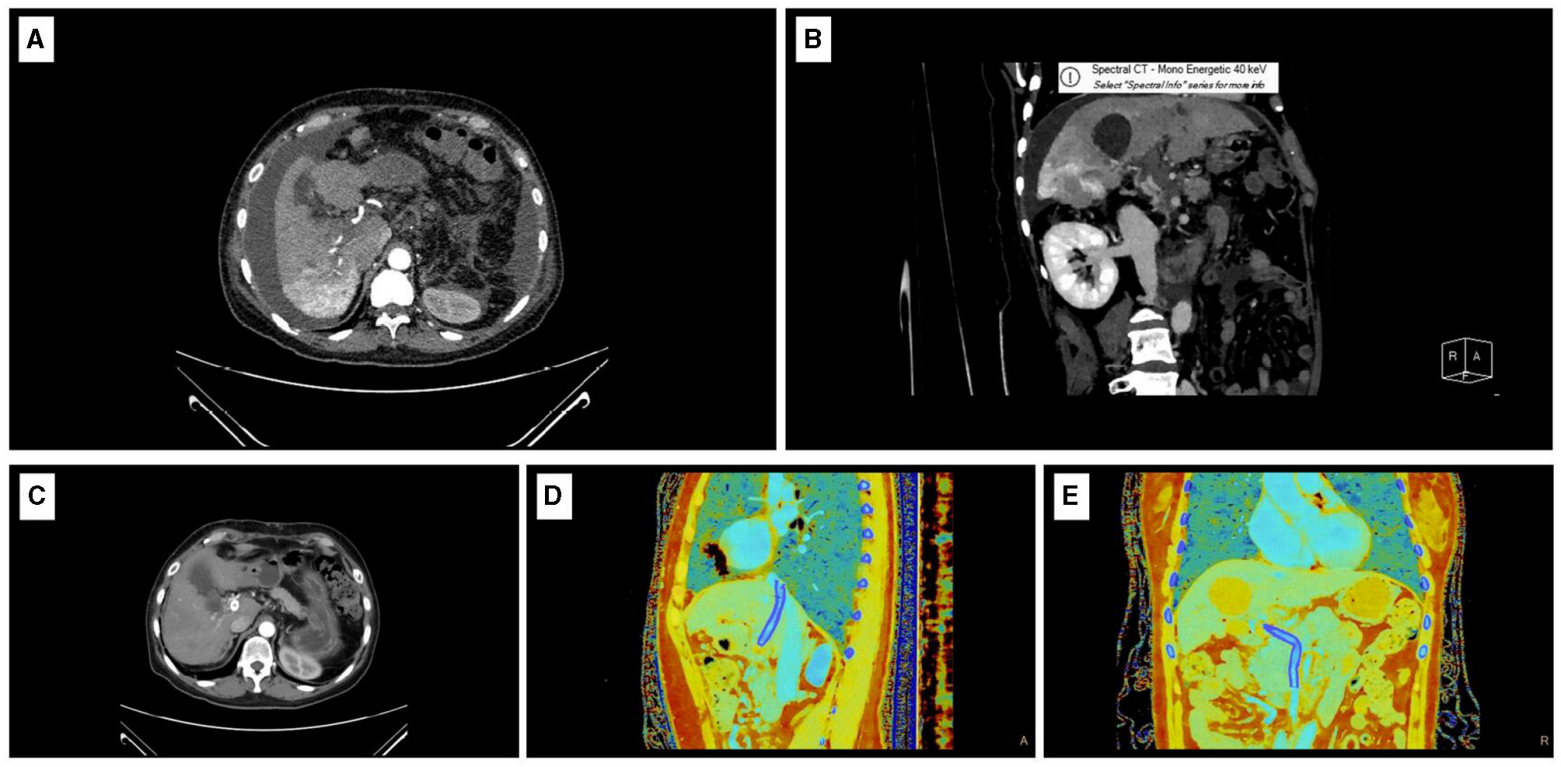
Imaging data. **(A)** Preoperative CT-enhanced arterial phase transverse image. **(B)** Preoperative CT-enhanced portal venous phase coronal image. **(C)** Postoperative CT-enhanced arterial phase transverse image. **(D)** Sagittal effective atomic number map on postoperative CT. **(E)** Coronal effective atomic number map on postoperative CT.

Preoperative imaging revealed abnormal perfusion in the liver. Computed tomography (CT) scans taken during the hepatic arterial phase showed high parenchymal enhancement in certain areas. These regions, known as transient hepatic attenuation differences (THADs) (35), appear as wedge-shaped zones with high blood flow during the hepatic arterial phase but become less dense in the venous and delayed phases (36). THADs can be associated with various conditions, including hepatocellular diseases, perihepatic diseases, portal vein obstructive diseases, liver tumors, hepatic inflammatory lesions, biliary tract diseases, and hepatic artery variations in the left hepatic lobe (37). In this case, the patient's abnormal hepatic perfusion was irregularly distributed in segments I, VI, and VII. The presence of localized abnormal perfusion changes warrants concern, as THADs may result from portal vein obstruction, potentially leading to temporary liver failure (35). The coexistence of malignant liver tumors and portal vein occlusion in this patient highlights the critical need to address these conditions. Diagnosis and treatment of liver diseases often involve identifying abnormal blood flow within the liver, and abnormal liver perfusion before surgery is commonly linked to PVT and HCC. Careful assessment of the patient's medical history and imaging results ruled out tumor spread to regions with irregular blood flow. The abnormal hepatic perfusion in this patient was attributed to PVT. TIPS placement has been shown to reduce portal pressures and improve systemic hemodynamics. However, issues with liver perfusion often remain unresolved (20, 38). Although significant improvements in abnormal perfusion were observed post-surgery, as evidenced by imaging (Figure 2C), ongoing monitoring is essential to ensure that no localized abnormal perfusion recurs. Follow-up examinations revealed notable enhancements in the patient's overall health and liver-kidney function, with no post-surgical complications and normalization of portal vein hemodynamic parameters. These findings provide valuable insights into managing complex cases requiring TIPS intervention.

The current TIPS program is advanced and sophisticated, allowing us to successfully complete a highly complex case and demonstrate our expertise in conducting TIPS procedures. Despite the positive outcome, this case certain limitations. The puncture of the mesenteric vein illustrates the technical challenges inherent to the procedure, necessitating precise surgical skills and advanced techniques. During the intervention, we encountered challenges, particularly with stent placement, but the patient's outcomes were favorable, underscoring the significance of experience. A holistic approach is essential in managing complex TIPS cases, with an emphasis on addressing complications and improving patient symptoms. These results validate our management strategy and emphasize the need for continuous improvement informed by new developments and successes.

The patient's favorable overall health status can be attributed to her insightful understanding of her medical condition, diligent adherence to prescribed treatments, and her occupation as a healthcare professional. Despite being diagnosed with malignant liver tumors, she exhibited a swift postoperative recovery. By the second day following the procedure, she demonstrated comfortable and pain-free mobility. She was discharged home after 2 weeks. A portal vein color Doppler ultrasound conducted one and a half months after the procedure revealed that the stent lumen was clear, with unrestricted blood flow and an average speed of 148 cm/s (2 January, 2024) (see Table 1). CT scans revealed stable stent placement, tumor stability, significant improvement in varices, and the disappearance of abdominal and pleural effusions. Effective atomic sequence images showed clear portal vein cavities (see Figures 2C–E), and the patient did not experience any bleeding or hepatic encephalopathy during follow-up care. She remains under regular follow-up care.

**Table 1:** Levels of serum liver function, renal function, portal vein diameter, and flow velocity.

**Group**	**AST**	**ALT**	**Cr**	**BUN**	**Internal diameter**	**Velocity**
	**(U/L)**	**(U/L)**	**(umol/L)**	**(mmol/L)**	**(mm)**	**(cm/s)**
**Before surgery**	125.08	93.06	37.47	7.61	5	-
**One month after surgery**	31.93	41.32	26.82	3.33	6.8	148
**Two months after surgery**	48.65	25.96	25.85	3.07	6.8	108

## 4 Conclusion

A complex case of liver cancer was described, involving gastric varices and portal venous cavernous transformation, previously treated with splenectomy in 2012. Preparing multiple surgical strategies can improve the success rate during the operation. Proficiency in various surgical techniques is important. In this case, the patient presented with portal vein occlusion and had undergone a splenectomy, along with poor vascular and tissue quality due to long-term liver disease. Conventional TIPS was not possible. Therefore, a modified access through TIPS using the mesenteric approach was used to achieve portal decompression. In complex cases, a detailed evaluation of the condition and proactive anticipation are necessary to handle complications effectively and prevent adverse events.

## Data availability statement

The raw data supporting the conclusions of this article will be made available by the authors, without undue reservation.

## Ethics statement

The studies involving humans were approved by the Ethics Committee, Gansu Provincial Hospital. The studies were conducted in accordance with the local legislation and institutional requirements. The participants provided their written informed consent to participate in this study. Written informed consent was obtained from the individual(s) for the publication of any potentially identifiable images or data included in this article.

## Author contributions

GS: Conceptualization, Investigation, Writing – original draft, Writing – review & editing, Formal analysis, Methodology, Supervision. JW: Formal analysis, Investigation, Writing – review & editing. BZ: Formal analysis, Investigation, Writing – review & editing. NZ: Formal analysis, Investigation, Writing – review & editing.
